# Multiple Brown Tumors Secondary to Parathyroid Carcinoma: A Challenging Diagnosis

**DOI:** 10.7759/cureus.31757

**Published:** 2022-11-21

**Authors:** Eugénia Silva, Rute Ferreira, Maria Helena Lourenço, Bernardo Marques, Sequeira Duarte

**Affiliations:** 1 Endocrinology, Diabetes and Metabolism, Egas Moniz Hospital, Lisbon, PRT; 2 Rheumatology, Egas Moniz Hospital, Lisbon, PRT

**Keywords:** endocrinology, metabolic bone disease, primary hyperparathyroidism, brown tumors, parathyroid carcinoma

## Abstract

Parathyroid carcinoma is an extremely rare endocrine neoplasm that accounts for less than 1% of the cases of primary hyperparathyroidism (PHPT). Continuous exposure to high levels of parathyroid hormone (PTH) induces an increase in bone remodeling and patients may present with osteitis fibrosa cystica, which is characterized by subperiosteal resorption of the phalanges, diffuse osteopenia, salt and pepper appearance of the skull, bone cysts, and brown tumors. Brown tumors occur in less than 5% of all patients with any form of hyperparathyroidism. Due to similar clinical, radiographic, and histological appearance, differential diagnosis of brown tumors includes primary and secondary bone tumors. We report a case of a 67-year-old female diagnosed with multiple osteolytic lesions initially thought to be bone metastasis of thyroid carcinoma. Further work-up led to the diagnosis of brown tumors due to parathyroid carcinoma. We want to emphasize the inclusion of osteitis fibrosa cystic in the differential diagnosis of osteolytic lesions and the need to perform serum calcium and PTH measurements when investigating these lesions.

## Introduction

Parathyroid carcinoma is an extremely rare endocrine neoplasm that accounts for less than 1% of the cases of primary hyperparathyroidism (PHPT). Nearly 90% of these tumors are hyperfunctioning tumors [1]. It occurs primarily as sporadic but also as part of genetic syndromes (familial isolated primary hyperparathyroidism, hyperparathyroidism-jaw tumor syndrome, and multiple endocrine neoplasia type 1 and type 2A syndromes). Usually, it follows an indolent clinical course, and its consequences are related to excessive secretion of PTH and hypercalcemia. Continuous exposure to high levels of PTH induces an increase in bone remodeling and patients can present with osteitis fibrosa cystica, characterized by subperiosteal resorption of the phalanges, diffuse osteopenia, salt and pepper appearance of the skull, bone cysts, and brown tumors [2]. Brown tumors are benign osteoclastic bone lesions that occur in less than 5% of all patients with any form of hyperparathyroidism. They mostly affect the pelvis, ribs, long bones, skull, and mandible [3]. Due to similar clinical, radiographic, and histological appearance, differential diagnosis of brown tumors includes secondary and primary bone tumors. We report a rare case of multiple brown tumors due to a parathyroid carcinoma that was initially suspicious for bone metastasis of thyroid carcinoma.

## Case presentation

We present the case of a 67-year-old female with no significant medical history, hospitalized due to bilateral SARS-CoV-2 pneumonia. In that context, she underwent a thoracic computed tomography (CT) scan that revealed multiple focal bone lesions on the ribs and the left shoulder blade which were suspicious for bone metastasis. When asked, the patient complained of asthenia and diffuse bone pain for nearly one year but never consulted a doctor. Laboratory evaluation showed hypercalcemia (10.6 mg/dL, reference range 8.5-10.1) and increased alkaline phosphatase levels (392 U/L, reference range 35-104). The patient was discharged after a few days and referred to an oncology appointment to proceed with the investigation.

Shortly after this hospitalization, she fell at home and had surgery due to a pathological diaphyseal fracture of the right humerus. Biopsy of the resected humerus was inconclusive but revealed histological features (such as multinucleated giant cells, some of them with hemosiderin deposits, in a fibroblastic stroma) that were consistent with a bone giant cell tumor (GCT).

**Figure 1:**
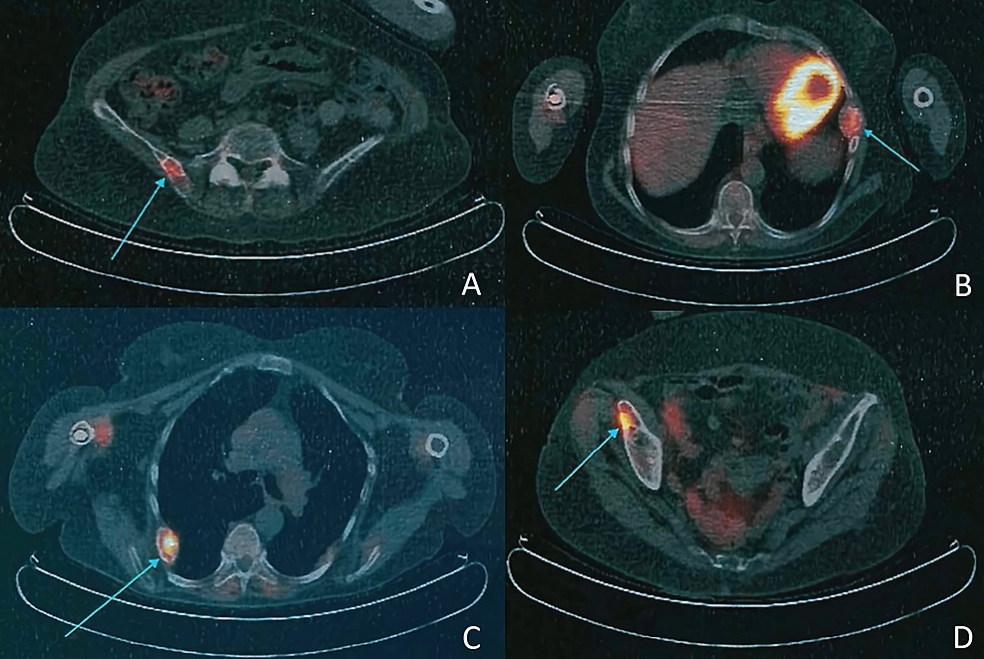
F-18-FDG positron emission tomography showing high focal uptake on the right iliac wing (A), rib cage (B, C), and right acetabulum (D).

Whole body CT showed a markedly heterogeneous thyroid nodule at the right lobe with 37 mm and several lytic lesions involving multiple bone elements in relation to extensive bone metastasis. It also revealed radiopaque bilateral kidney stones. The F-18-FDG positron emission tomography (18F-FDG PET) showed high focal uptake compatible with malignant neoplastic etiology at those bone lesions, but no high uptake at the thyroid nodule (Figures 1A-1D).

The patient was submitted to cervical ultrasonography (US) that revealed a 27-mm hypoechoic solid nodule on the inferior right thyroid lobe. Fine needle aspiration cytology was suspicious for follicular neoplasm (Bethesda IV category) and a total thyroidectomy was performed. The histological study revealed adenomatous hyperplasia, thyroiditis foci, and a parathyroid adenoma. The patient was referred to an Endocrinology department for further follow-up.

She maintained bone pain and was already on medication with paracetamol 1,000mg three times a day, gabapentin 600mg twice daily, and transdermal fentanyl 75mcg/h replaced every 72h. Laboratory tests showed hypercalcemia and elevated PTH, compatible with PHPT (serum calcium 11.5 mg/dL and PTH 809.0 pg/mL; Table 1).

**Table 1:** Laboratory parameters in the first Endocrinology appointment and six months after subtotal parathyroidectomy

Laboratory Parameter	First Endocrinology appointment	Six months after subtotal parathyroidectomy	Reference Range
Albumin-corrected serum calcium (mg/dL)	11.5	8.9	8.9-10.2
Phosphate (mg/dL)	3.2	3.6	2.5-4.5
Parathyroid hormone (pg/mL)	809.0	28.9	15.0-65.0
Magnesium (mEq/L)	1.9	2.2	1.6 – 2.4
25-hydroxyvitamin D (mmol/L)	<20.0	97.0	75.0-250.0
Creatinine (mg/dL)	1.4	1.6	0.5 – 0.9
Urinary Calcium (mg/24h)	34.0	-	100.0-300.0
Alkaline phosphatase (U/l)	402.0	97.0	35.0-104.0

**Figure 2:**
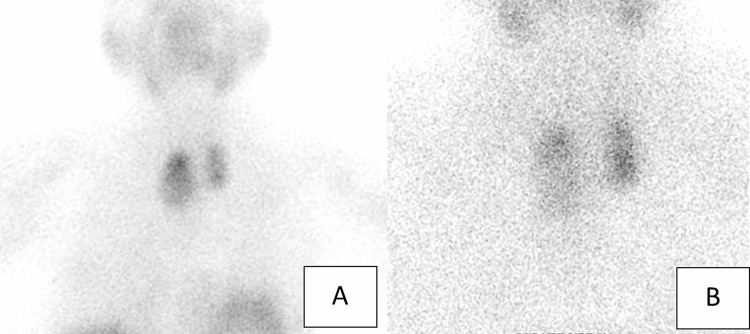
99mTc-sestamibi parathyroid scintigraphy of the neck obtained at the baseline (A) and two hours after (B), showing intense uptake on both sides consistent with hyperactive inferior left and right parathyroid glands.

The cervical US revealed a 27.5 mm hypoechoic solid nodule below the left thyroid surgical site and a 16.9mm isoechoic solid nodule below the right thyroid surgical site, suggestive of parathyroid glands. The sestamibi scintigraphy demonstrated increased uptake at the inferior left and right parathyroid glands (Figures 2A, 2B).

 

**Figure 3:**
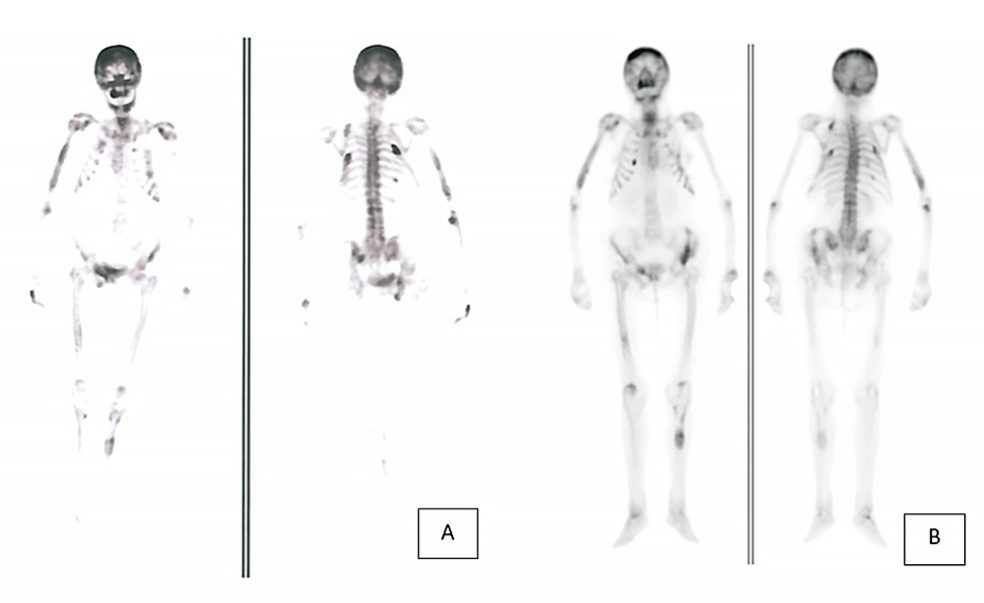
A bone scan previous (A) and six months (B)after treatment showing multiple brown tumors located on the skull, face, right acetabulum, right pubis, middle third of the left tibia, bilateral femur, rib cage, and shoulder blades.

At 99mTcHDP skeletal scintigraphy, there was diffusely increased uptake in the skull, face, both femoral diaphysis and right humerus diaphysis, and a focal increased uptake (due to reactive osteoblastic bones surrounding the lesion) on the rib cage, shoulder blades, right pubis, right acetabulum, and left tibia (this one being a lytic lesion) (Figure 3A).

 

Multiple brown tumors were diagnosed based on the histopathological findings (multinucleated giant cells, some of them with hemosiderin deposits, in a fibroblastic stroma) and the history of PHPT. The scintigraphic features of metabolic bone disease may also suggest PHPT and thus brown tumors.

The patient was submitted to a subtotal parathyroidectomy with the sacrifice of the right recurrent nerve due to tumor invasion. The final histological diagnosis was parathyroid carcinoma (in both excised glands).

A genetic study found no germline mutations of CASR, CDC73, CDKN1B, GCM2, GNA11, MEN1, and RET genes. Six months after surgery, the patient maintains therapy with calcium carbonate 1,500 mg twice daily and calcitriol 0.25 mcg once daily. The last laboratory evaluation revealed normalization of serum calcium and PTH levels (serum calcium 8.9 mg/dL and PTH 28.9 pg/mL; Table 1). The 18F-FDG PET and the bone scintigraphy, six months after surgery, show maintenance of the bone lesions (Figure 3B).

The patient shows improvement in tiredness and bone pain, and the analgesic therapy is gradually being reduced. Currently, she maintains therapy with gabapentin 100 mg twice daily and paracetamol 1,000mg once daily.

## Discussion

We described a rare case of multiple brown tumors due to PHPT caused by parathyroid carcinoma. The diagnosis of multiple brown tumors is supported by the finding of giant cells in the humerus histopathology along with the presence of hyperparathyroidism. In the context of PHPT, giant cell lesions are considered reparative granulomas and not potentially malignant neoplasms as true GCT. Normalization of serum calcium, alkaline phosphatase, and PTH levels after parathyroidectomy along with the maintenance of the bone lesions allow us to exclude the hypothesis of bone metastasis of parathyroid carcinoma. The absence of another primary tumor on PET and CT makes the hypothesis of bone metastasis from another tumor unlikely.

Brown tumors are part of the GCT family. They usually develop in the fifth decade of life and predominantly affect females [4,5]. They may cause swelling, pathological fracture, and bone pain due to high bone turnover associated with long-standing hyperparathyroidism. Histology of these lesions typically demonstrates increased fibroblastic proliferation, multinucleated giant cells, restorative granulomas, areas of cystic degeneration, hemorrhage, and macrophages with hemosiderin [6]. 

As shown in this case, a histological analysis may not guarantee a diagnosis, as brown tumors may be indistinguishable from primary or metastatic bone tumors, especially from the giant-cell variant. Some bone GCTs occur in the setting of Paget disease, a rare disease characterized by disordered bone turnover and high levels of alkaline phosphatase. Brown tumors may also mimic skeletal metastasis on bone scintigraphy or 18F-FDG PET/CT. False positive areas of increased FDG uptake might be associated with the presence of giant cells and macrophage glucose metabolism and should not lead to an immediate diagnosis of metastases [7].

Although parathyroid adenomas are the most common cause of brown tumors due to PHPT, clinicians must be aware of the possibility of the diagnosis of parathyroid carcinoma, as osteitis fibrosa cystica and other radiological signs of skeletal disease are much more common in that case (44%-91% in parathyroid carcinoma versus less than 5% in benign lesions) [8].

Clinical and biochemical presentations are usually more severe in carcinomas than in other PHPT causes and markedly elevated serum calcium and PTH concentration levels should raise the suspicion index (levels of serum calcium above 14 mg/dL or 3-4 mg/dL above the upper limit of normal and PTH ≥4 times above the upper limit of normal) [9]. Alkaline phosphatase is also higher in patients with parathyroid carcinoma.

The recommended treatment for brown tumors is to treat hyperparathyroidism. After that, bone lesions tend to regress, but in some cases, maintenance or an increase of the tumors can occur [10]. Surgical excision of these tumors is mostly reserved for symptomatic patients, pathological fractures, broad cortical involvement, or unexpected growth despite the therapy.

## Conclusions

Brown tumors can be the first sign of PHPT but have become a rarity and for that reason may not be considered in the differential diagnosis of osteolytic lesions. We want to emphasize the inclusion of osteitis fibrosa cystica in the differential diagnosis of osteolytic lesions and the importance of serum calcium and PTH measurements when investigating these lesions.
